# Case Report: Exceptional Response to Second Line Temozolomide Therapy in a Patient With Metastatic Adrenocortical Carcinoma

**DOI:** 10.3389/fendo.2021.674039

**Published:** 2021-04-22

**Authors:** Deborah Cosentini, Antonella Turla, Ornella Carminati, Salvatore Grisanti, Vittorio Domenico Ferrari, Marta Laganà, Giovanni Rosti, Sandra Sigala, Alfredo Berruti

**Affiliations:** ^1^ Medical Oncology Unit, Azienda Socio Sanitaria Territoriale (ASST) Spedali Civili, Department of Medical and Surgical Specialties, Radiological Sciences, and Public Health, University of Brescia, Brescia, Italy; ^2^ Medical Oncology, Department of Oncology and Hematology, Azienda Unità Sanitaria Locale (AUSL), Romagna, Ravenna, Italy; ^3^ Medical Oncology, Fondazione IRCCS Policlinico San Matteo, Pavia, Italy; ^4^ Section of Pharmacology, Department of Molecular and Translational Medicine, University of Brescia, Brescia, Italy

**Keywords:** adrenal tumor, alkylating drug, progesterone, treatment, ACC

## Abstract

**Background:**

In a recently published retrospective case series, Temozolomide was found active as second line approach in advanced ACC patients. The disease control rate obtained, however, was short-lived. We report here an ACC patient with extensive metastatic disease who obtained a remarkable long lasting response with this alkylating agent.

**Case Presentation:**

a 22-year-old female patient with ACC presented at our Medical Oncology Department in poor general condition due the presence of extensive metastatic pulmonary involvement. The disease had progressed to etoposide, doxorubicin and cisplatin plus mitotane therapy. Second line temozolomide therapy was prescribed leading to a progressive improvement of patient general conditions. The disease restaging after 12 cycles revealed a complete response of lung lesions and the patient was free from progression for 14+ months.

**Conclusion:**

Temozolomide therapy could be exceptionally efficacious in the management of ACC patients. The molecular mechanisms of sensitivity and resistance to this drug should be carefully studied, in order to select the patients destined to obtain a significant clinical benefit to the drug.

## Background

Adrenocortical carcinoma (ACC) is a rare and aggressive tumor with an incidence of 0.7-2 new cases per million populations per year (1). Surgery is the mainstay of therapy for localized diseases and patients with moderate to high risk of disease relapse and death are addressed to adjuvant mitotane therapy (2). The standard systemic treatment for advanced/metastatic tumors, not amenable to surgical resection, is the combination of etoposide, doxorubicin and cisplatin plus mitotane (EDP-M) (3). The results of a large multicenter randomized clinical trial have demonstrated that the efficacy of this regimen is limited with a disease response of about 25% and a median overall survival of 14 months (4). However, a single Institution case series, in which surgery of residual disease after EDP-M was systematically performed when feasible, revealed that few patients (7%) can obtain a pathological complete response and a long term disease control (5). These data suggest that EDP-M scheme can be sometimes very efficacious. Few options are available for patients with disease progression to EDP-M. Gemcitabine plus metronomic capecitabine is a second line approach recommended by international guidelines (1), but the efficacy of this regimen is modest (6). Several target therapies substantially failed to demonstrate activity in ACC (7, 8) and the role of immunotherapy is currently under investigation (9).

In a recently conducted retrospective study, single agent temozolomide was found active as second line approach in advanced ACC patients with a response rate observed in 20% of patients. The disease control rate obtained, however, was short-lived (10).

Here, we presented the case of a 22-year-old female patient with advanced ACC reporting a dramatic disease response to temozolomide therapy.

## Case Presentation

A 22-year-old woman from Sicily presented in August 2019 at the Medical Oncology Unit of Santa Maria delle Croci hospital in Ravenna, Italy, with severe hypertension, hirsutism, oligomenorrhea, and epigastric pain. A CT scan showed a huge left adrenal mass (100x110mm) and multiple lung metastasis. Hormonal assessment revealed elevated plasma and urinary cortisol levels and elevated androgen levels. The patient’s general conditions were compromised due to a severe Cushing’s syndrome. A tumor biopsy was performed confirming the ACC diagnosis. The patient was immediately addressed to first line chemotherapy with the EDP-M regimen, administered in association with metyrapone, to rapidly obtain a control of hormone hypersecretion (11). A minimal disease response of lung metastases and no change of the primary tumor lesion was obtained after 2 chemotherapy cycles. The patient general conditions, however, consistently improved. Antineoplastic therapy was continued and, from the 3^rd^ cycles onwards, mitotane serum levels had attained the therapeutic interval, ranging between 14.5 and 20.1 ng/ml. Unfortunately, a huge disease progression was observed at TC restaging after 5 cycles.

The patient was then referred to the Medical Oncology Unit in Brescia in January 2020. On admission her general conditions were poor (ECOG performance status 3). She suffered from uncontrolled pain, nausea, asthenia and dyspnea. Oxygen saturation was low (SpO2 88%), so continuous oxygen therapy was instituted.

**Figure 1:**
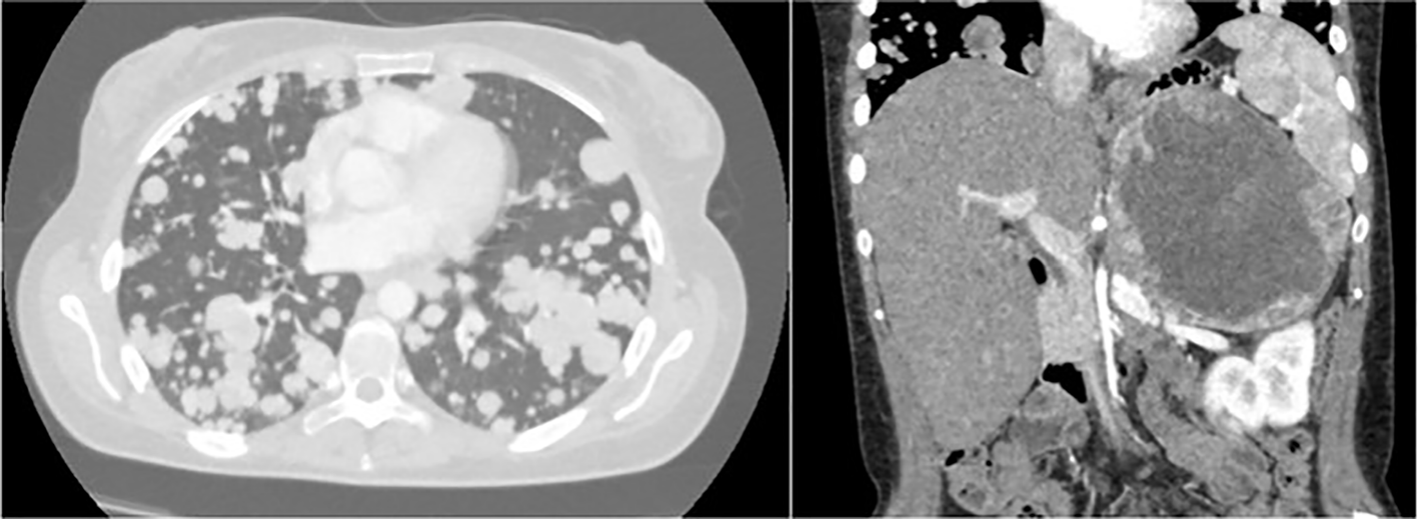
Multiple pulmonary metastases and adrenal mass detected by CT Scan (January 2020).

The CT scan confirmed the voluminous left adrenal mass (160x110 mm in size) infiltrating the left kidney and revealed a dramatic disease progression in lung with multiple lesions affecting both hemithoraxes (**Figure 1**).

Due her serious conditions we deemed the patient not eligible to further intravenous chemotherapy. She was then discharged and entrusted to home palliative care in Sicily with the prescription of oral temozolomide at the dose of 200 mg/m2/die given for 5 consecutive days every 28 days and daily. Megestrol acetate 160 mg every day was also concomitantly prescribed to support appetite and patient kenesthesis. Mitotane was continued.

**Figure 2:**
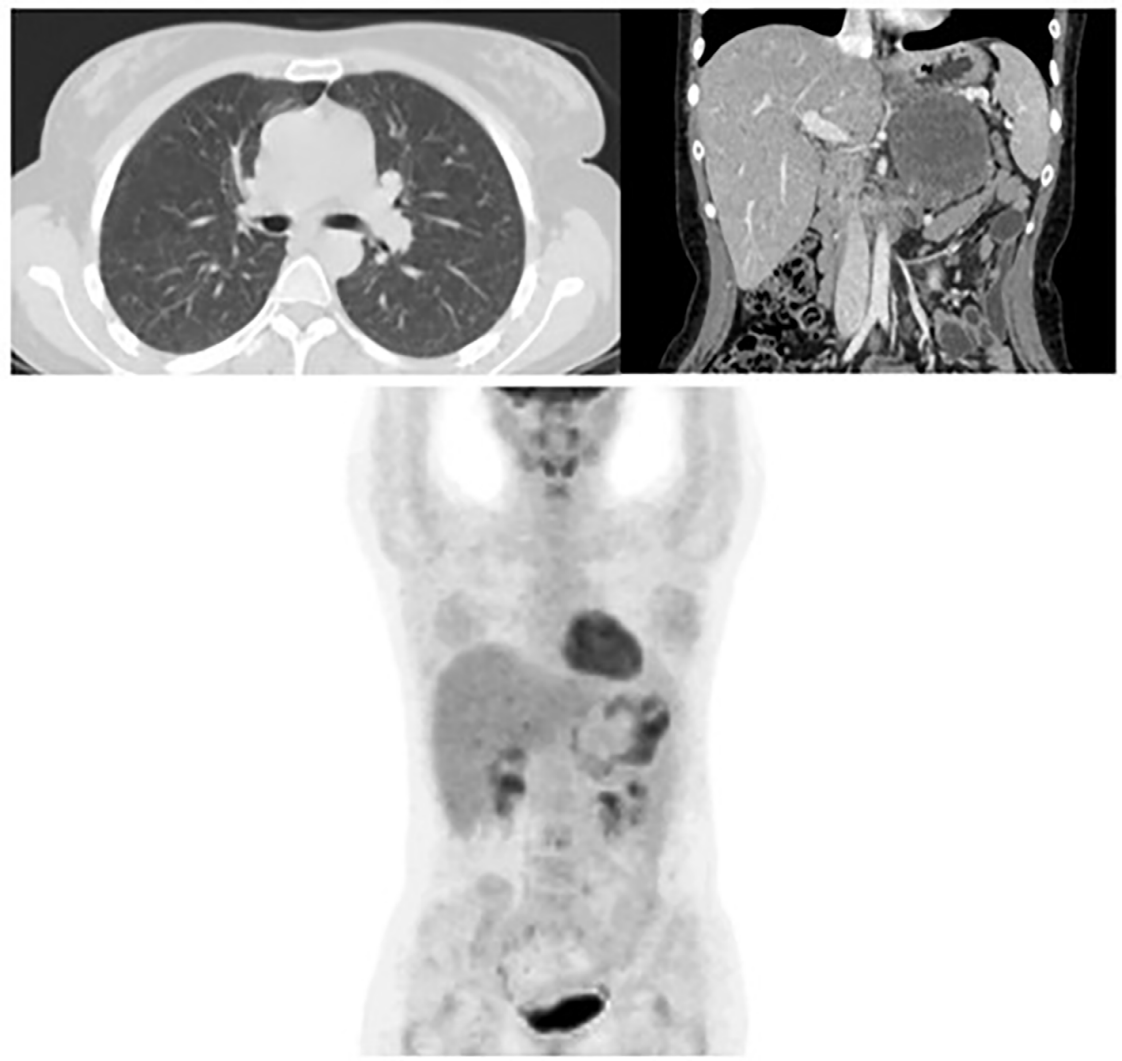
Complete response of the bilateral lung metastases and reduction of the primary adrenal lesion at the CT Scan after 8 cycles of temozolomide. FDG PET confirming the complete response of lung lesions and a small peripheral area of FDG uptake at the residual adrenal mass. (September 2020).

Progressively, the patient general conditions improved with a normalization of oxygen saturation and pain control. Temozolomide therapy was well tolerated without any relevant toxicity. After 2 months her quality of life returned to normal and megestrol acetate was interrupted while continuing both temozolomide and mitotane therapies. The CT restaging performed in March 2020 showed a partial response of lung metastases with a minimal response of the adrenal mass. The extent of lung disease further decreased at a subsequent CT in June 2020. In September the patient returned to Brescia for a follow-up visit. A CT scan revealed a complete disease response of lung metastases and a partial response of the abdominal lesion with dimension reduction (60 mm) and necrosis increase (**Figure 2**). The FDG PET scan confirmed the complete response of the lung lesions and, as regard the abdominal mass, a small peripheral uptake was described with an extensive central area with no FDG (fluorodesoxyglucose) uptake (necrosis) (**Figure 2**).

The last CT scan (December 2020) revealed a stable disease with respect to the previous control in September. At the last follow-up examination, on February 25^th^, the patient general conditions were excellent (ECOG performance status 0). She was still on Temozolomide treatment (12 total cycles) along with mitotane and was free from progression for 14 months.

A Next Generation Sequencing (NGS) analysis, with a wide panel of genes (genic variant in hotspot regions of 35 genes -AKT1, ALK, AR, BRAF, CDK4, CTNNB1, DDR2, EGFR, ERBB2, ERBB3, ERBB4, ESR1, FGFR2, FGFR3, GNA11, GNAQ, HRAS, IDH1, IDH2, JAK1, JAK2, JAK3, KIT, KRAS, MAP2K1, MAP2K2, MET, MTOR, NRAS, PDGFRA, PIK3CA, RAF1, RET, ROS1, SMO; amplification of 19 genes- ALK, AR, BRAF, CCND1, CDK4, CDK6, EGFR, ERBB2, FGFR1, FGFR2, FGFR3, FGFR4, KIT, KRAS, MET, MYC, MYCN, PDGFRA, PIK3CA; rearrangements of 23 genes- ABL1, AKT3, ALK, AXL, BRAF, ERG, ETV1, ETV4, ETV5, EGFR, ERBB2, FGFR1, FGFR2, FGFR3, MET, NTRK1, NTRK2, NTRK3, FDGFRA, PPARG, RAF1, RET, ROS1) was performed on biopsy tumor samples at diagnosis. A genic variant in CTNN1B exon 3 (p.Met14Leu) was found (**Table 1**). This variant has never been published as pathogenetic in literature and it is not present in ClinVar, whereas it is categorized as VUS (variance of uncertain significance) in the VARSOME database. O6-methylguanine-DNA methyl-transferase (MGMT) and progesterone receptor (PgR) expression could not be assessed, due to insufficient tumor materials.

**Table 1:** Genic variant detected by NGS.

Chromosome	Gene	Genic region	Proteic variant (cDNA)	Variant type	Coverage	Allelic frequency	Reference sequence
3	CTNN1B	Exon 3	p.Met14Leu (c.40A>C)	Nucleotide sustitution	1759	39.9%	NM_001098210.1 (GenBank)

## Discussion

Chemotherapy + mitotane (EDP-M) is modestly active as first first-line approach in the management of patients with advanced/metastatic ACC. At disease progression to EDP-M, the further administration of cytotoxic drugs gave disappointing results. At the best of our knowledge, this is the first ACC case that has obtained a dramatic response with a second-line chemotherapy. This result is even more relevant if we consider that the patient in question had an extensive disease burden and her performance status was very low. The latter condition is known to be an independent factor of poor efficacy of systemic antineoplastic treatments in general and ACC in particular (1).

It is certainly not clear why second-line temozolomide was particularly effective in this case. The drug was found to be able to induce a significant cytotoxic effect in ACC cells *in vitro* (12). However, in a recent Italian case series of pre-treated ACC patients, it proved to be moderately active with a disease response rate observed in 20% of treated cases, but poorly effective, as disease control was short lived (3.5 months on average) (10).

The addition of megestrol acetate for palliation may have contributed in the first weeks to support the patient’s performance status and favored the cytotoxic effect of temozolomide. A recent paper by our group has in fact demonstrated an antineoplastic effect of progesterone in ACC cell lines and primary ACC cultures (13). Unfortunately, due to insufficient tissue material, it was not possible to test the expression of the PgR and MGMT in the primary tumor, which are known predictors of efficacy of megestrol acetate and temozolomide, respectively. The NGS performed failed to provide a potentially valuable predictive parameter.

In conclusion, the exceptional and long lasting disease response obtained with temozolomide in this ACC patient, suggests that this drug can be very efficacious in this setting. Temozolomide deserve to be further tested in ACC patients with the aim of identifying predictive factors of efficacy in order to select the patients destined to obtain a significant clinical benefit to the drug.

## Data Availability Statement

The raw data supporting the conclusions of this article will be made available by the authors, without undue reservation.

## Ethics Statement

The studies involving human participants were reviewed and approved by Brescia ethics committee. The patients/participants provided their written informed consent to participate in this study. Written informed consent was obtained from the individual(s) for the publication of any potentially identifiable images or data included in this article.

## Author Contributions

AB conceived the idea of this manuscript. OC, VF, GR, and AB clinically followed the patient. DC, AT, and ML collected and interpreted the patient clinical data and wrote the manuscript. All authors contributed to the article and approved the submitted version.

## Funding

This study was supported in part by FIRM onlus, Cremona, Italy and by Associazione Italiana per la Ricerca contro il Cancro (AIRC), IG: 14411.

## Conflict of Interest

The authors declare that the research was conducted in the absence of any commercial or financial relationships that could be construed as a potential conflict of interest.
